# RNA Aptamer Probes as Optical Imaging Agents for the Detection of Amyloid Plaques

**DOI:** 10.1371/journal.pone.0089901

**Published:** 2014-02-26

**Authors:** Christian T. Farrar, Christopher M. William, Eloise Hudry, Tadafumi Hashimoto, Bradley T. Hyman

**Affiliations:** 1 Athinoula A. Martinos Center for Biomedical Imaging, Department of Radiology, Massachusetts General Hospital and Harvard Medical School, Charlestown, Massachusetts, United States of America; 2 Department of Pathology, Massachusetts General Hospital and Harvard Medical School, Charlestown, Massachusetts, United States of America; 3 Department of Neurology, Massachusetts General Hospital and Harvard Medical School, Charlestown, Massachusetts, United States of America; Massachusetts General Hospital/Harvard Medical School, United States of America

## Abstract

Optical imaging using multiphoton microscopy and whole body near infrared imaging has become a routine part of biomedical research. However, optical imaging methods rely on the availability of either small molecule reporters or genetically encoded fluorescent proteins, which are challenging and time consuming to develop. While directly labeled antibodies can also be used as imaging agents, antibodies are species specific, can typically not be tagged with multiple fluorescent reporters without interfering with target binding, and are bioactive, almost always eliciting a biological response and thereby influencing the process that is being studied. We examined the possibility of developing highly specific and sensitive optical imaging agents using aptamer technology. We developed a fluorescently tagged anti-Aβ RNA aptamer, β55, which binds amyloid plaques in both *ex vivo* human Alzheimer’s disease brain tissue and *in vivo* APP/PS1 transgenic mice. Diffuse β55 positive halos, attributed to oligomeric Aβ, were observed surrounding the methoxy-XO4 positive plaque cores. Dot blots of synthetic Aβ aggregates provide further evidence that β55 binds both fibrillar and non-fibrillar Aβ. The high binding affinity, the ease of probe development, and the ability to incorporate multiple and multimodal imaging reporters suggest that RNA aptamers may have complementary and perhaps advantageous properties compared to conventional optical imaging probes and reporters.

## Introduction

An emerging class of novel molecular probes based on RNA or DNA aptamers may offer significant advantages as both therapeutic and diagnostic imaging agents [1]–[4]. Very large RΝΑ libraries can be rapidly screened to identify sequences that bind a given molecule with high affinity using Selective Evolution of Ligands by EXponential enrichment (SELEX) [5], [6]. The affinities and specificities of RNA aptamers are comparable to or even better than those of antibodies [1], [7]. Aptamers can fold back into their natural conformation after denaturation and are stable in the reducing environment of the cell [3]. Aptamers are much smaller than antibodies, thereby improving their biodistribution [3], [8]. Aptamers can be delivered to cells using viral vectors [4], [9]. Aptamers can also be made by chemical synthesis, which allows for tailor-made modifications and avoids biological contamination. Imaging agents can be easily incorporated into aptamers using labeled nucleotides, providing the potential for multiplexing and for tuning reagents to the imaging platform. For example, fluorescein-tagged nucleotides can be used for 2-photon imaging, Cy5-tagged nucleotides for near infrared imaging, ^18^F-labled nucleotides for positron emission tomography (PET) imaging, and ^19^F-labeled nucleotides for ^19^F magnetic resonance imaging (MRI). Aptamers may also provide powerful tools for developing therapeutic agents [3], [4]. Aptamers have even been selected that cross the blood-brain barrier [10]. Finally, aptamers typically have low or no immunogenicity [3], [11]. As a test of the idea that aptamers could be used as a new *in vivo* optical imaging tool, we have investigated the use of a fluorescently labeled anti-Aβ aptamer for imaging both amyloid plaques and oligomeric Aβ in Alzheimer’s disease (AD) mouse models.

Increasing evidence suggests that soluble oligomeric Aβ is synaptotoxic and plays a central role in the early pathogenesis of AD [12], [13]. Studies in neuronal cultures and organotypic slices demonstrated that soluble forms of Aβ induce synaptic changes and dendritic spine loss and are toxic to mature central nervous system neurons [14]–[16]. Shankar *et al* demonstrated that Aβ dimers, but not insoluble plaque cores, impaired long-term potentiation, lowered the threshold for induction of long-term depression, and reduced dendritic spine density in normal mouse hippocampus [17]. Similarly, the presence of a 56-kDa Aβ assembly has been correlated with memory loss in Tg2576 transgenic mouse models of AD [18], [19], while Tris-buffered saline (TBS) soluble Aβ from Alzheimer’s disease brain has been shown to disrupt the memory of a learned behavior in normal rats [17]. Koffie *et al* demonstrated that oligomeric Aβ, present in a halo surrounding dense core plaques, is associated with postsynaptic densities and correlates with excitatory synapse loss near amyloid plaques [20], [21]. Finally, recent studies of transgenic mice that co-express mutant forms of amyloid precursor protein (APP) and tau have demonstrated that oligomeric Aβ accumulation, but not total amyloid plaque burden, correlates with neuronal loss and inflammatory response [22]. These studies suggest that amyloid plaques serve as reservoirs of oligomeric Aβ and that oligomeric Aβ is synaptotoxic. Given this evidence of a direct role of Aβ oligomers in AD, there is a great need for new reagents capable of detecting not only mature amyloid plaques but also oligomeric forms of Aβ.

A large number of antibodies that recognize a variety of epitopes of different Aβ assemblies, including low molecular weight Aβ oligomers, have been developed and used extensively in *ex vivo* studies [23]–[31]. However, the use of antibodies for *in vivo* studies is complicated by the plaque clearance that is induced by anti-Aβ antibodies [32]. In addition, the incorporation of multiple optical labels, to provide for increased detection sensitivity, typically significantly reduces the antibody binding affinity. A small molecule positron emission tomography (PET) tracer for imaging amyloid plaques that crosses the blood-brain barrier (BBB), Pittsburgh Compound-B (PiB), was developed and has been successfully used in many clinical studies [33]. Similarly, a small molecule fluorescent derivative of Congo Red, methoxy-XO4, has been extensively used in optical studies of AD transgenic mouse models [34]. However, while these small molecule amyloid probes do cross the BBB and are able to detect fibrillar Aβ, they do not detect oligomeric Aβ. If oligomeric Aβ burden is more directly related to synaptic toxicity and the risk of developing AD, a probe that bound Aβ oligomers might provide a more specific reporter for the risk of developing AD. Approaches to image oligomeric Aβ, even if only in experimental models, may prove important to inform our understanding of the pathobiology of these species *in vitro* and *in vivo*.

A novel RNA aptamer, β55, generated from a SELEX screen against monomeric Aβ_1–40_ was previously shown to bind synthetic amyloid fibrils [35]. Similar SELEX screens against covalently stabilized Aβ trimers [36] and against Aβ_1–40_ conjugated to colloidal gold nanoparticles [37] have been performed. More recently, a DNA aptamer selected against α-synuclein was shown to also bind oligomeric Aβ [38]. However, to date no *ex vivo* stains of human AD brain tissue or *in vivo* mouse studies in amyloid-laden transgenic AD models have been performed with aptamer probes. Here we demonstrate for the first time the ability of β55 to bind amyloid plaques in *ex vivo* human AD brain tissue and in live transgenic mouse models of AD. Furthermore, we demonstrate that β55 binds Aβ oligomers and is able to visualize the oligomeric halo surrounding the dense cores of amyloid plaques. This is the first use of an aptamer as an *in vivo* optical imaging probe.

## Materials and Methods

### Synthesis of Labeled RNA Aptamers

Double-stranded DNA template (132 bp) was synthesized by polymerase chain reaction of the 76 mer 5′ ends of β55 and its reverse complement (Operon Biotechnologies, Huntsville, AL), which contain a 20 bp overlap. The DNA sequence (Table 1) included a 25 mer T7 RNA polymerase primer. Imaging probes were introduced into the aptamer by transcription of the DNA template with T7 RNA polymerase and either biotin-labeled uracil (Roche Diagnostics, Indianapolis, IN), for *ex vivo* studies, or fluorescein-labeled uracil (Roche Diagnostics, Indianapolis, IN), for *in vivo* studies, using the Hi-Scribe RNA transcription kit (New England BioLabs, Ipswich, MA). RNA was purified twice on Mini Quick-Spin RNA columns (Roche Diagnostics, Indianapolis, IN) to ensure complete removal of unincorporated nucleotides. The RNA sequence length was characterized both by running the RNA on a denaturing gel and an Agilent 2100 Bioanalyzer using a small RNA kit (Agilent Technologies, Santa Clara, CA). Both methods observed an RNA length of just over 100 nucleotides consistent with the expected length of 107 nucleotides. RNA concentrations were determined from the absorbance measured at 260 nm using a Biophotometer (Eppendorf, Hauppauge, New York). Typical RNA concentrations after purification ranged between 25–35 µM.

**Table 1:** DNA sequence for the β55 aptamer including the T7 polymerase primer, highlighted in bold text.

5′-**GCG TAA TAC GAC TCA CTA TAG GGC G**GG GAA TTC GAG CTC GGT ACC TTT ACC GTA AGG CCT GTC TTC GTT TGA CAG CGG CTT GTT GAC CCT CAC ACT TTG TAC CTG CTG CCA ACT GCA GGC ATG CAA GCT TGG-3′

### Aptamer Staining of *ex vivo* AD Brain Tissue

Frozen brain tissue from AD subjects was obtained from the Massachusetts Alzheimer’s Disease Research Center (http://madrc.mgh.harvard.edu/) tissue bank. All the study subjects or their next-of-kin gave written informed consent for the brain donation at their respective institutions. Tissue was sectioned on a cryostat at a thickness of 10 µm and fixed for 10 minutes in 4% paraformaldehyde followed by overnight incubation at 4°C with either biotinylated β55 aptamer or its reverse complement, β55rc, used as a control probe. Probes were visualized by reacting with avidin binding complex followed by tyramide signal amplification using AlexaFluor 488 tyramide (Invitrogen, Carlsbad, CA). For slides co-stained with Thioflavin-S, AlexaFluor 594 tyramide (Invitrogen, Carlsbad, CA) was used for visualizing β55 positive plaques in the red channel. Slides were coverslipped and imaged on either an Olympus BX51 fluorescence microscope (Olympus, Tokyo, Japan) or a Zeiss LSM 510 confocal microscope (Carl Zeiss MicroImaging, Jena, Germany).

### Dot and Western Blot of Synthetic Aβ Aggregates

1.1 µM of synthetic Aβ_1–40_ and Aβ_1–42_ (Peptide Institute, Osaka, Japan) were incubated at 37°C for 3 days with gentle shaking. 4 µl each of Aβ_1–40_ and Aβ_1–42_ were blotted onto nitrocellulose paper and allowed to dry. The dot blot was then stained with biotinylated β55. β55 was visualized by secondary staining with streptavidin IRDye 700DX (Rockland Immunochemicals, Gilbertsville, PA) and imaged on an Odyssey infrared imaging system (LI-COR Biosciences, Lincoln, NB). Western blots of the synthetic Aβ reaction mixtures were also performed to identify the Aβ species present in each reaction mixture. Western blots were stained with 6E10 antibody (1∶1000), which is reactive to amino acid residues 3–8 of Aβ. 6E10 was visualized by secondary staining with anti-mouse IgG antibody conjugated to IRDye800 (Rockland Immunochemicals, Gilbertsville, PA) and imaged on an Odyssey infrared imaging system (LI-COR Biosciences, Lincoln, NB).

### In vivo 2-photon Imaging of APP/PS1 Transgenic Mice

*In vivo* multiphoton images of optical fluorophores were obtained in real time using an Olympus Fluoview 1000 MPE with prechirp optics mounted on an Olympus BX61WI upright microscope. APP/PS1 transgenic mice which coexpress mutant alleles of amyloid precursor protein (APP) and presenilin (strain B6C3-Tg[APPswe, PSEN1dE9]85Dbo/J, Jackson Laboratory, Bar Harbor, ME) [39] were used for all imaging experiments. After craniotomy, 20 µg of fluorescein labeled aptamer probe (either β55 or β55rc) was topically applied to the mouse brain for 30 minutes. The surface of the brain was then flushed with artificial cerebral spinal fluid, followed by placement of the coverslip window. A wax ring was placed on the edges of the coverslip covering the craniotomy window and was filled with distilled water to maximize contact between water and an Olympus 20× dipping objective (numerical aperture, 0.95). A mode-locked titanium/sapphire laser (MaiTai; Spectra-Physics) generated two-photon fluorescence with 800-nm excitation, and detectors containing three photomultiplier tubes (Hamamatsu) collected emitted light in the ranges of 380–480, 500–540, and 560–650 nm (4). Average power reaching brain tissue in each experiment ranged from 20 to 60 mW. Images were acquired between 100 and 500 µm below the surface of the brain. Images were acquired approximately 1 and 24 hours after topical application of the aptamer probe. All animal experiments were approved by the Massachusetts General Hospital Subcommittee on Research Animal Care (SRAC protocol # 2004N000092) and conformed to national and institutional guidelines.

### Determination of Plaque Contrast-to-noise Ratio

Plaque regions-of-interest (ROI) were determined by thresholding the *in vivo* images to signals greater than two standard deviations above the background signal intensity. The plaque contrast-to-noise ratio (CNR) was calculated from the difference between the plaque and background signal-to-noise ratios (SNR). The gold standard used for the identification and counting of the number of amyloid plaques was the presence of methoxy-XO4 staining.

## Results

**Figure 1:**
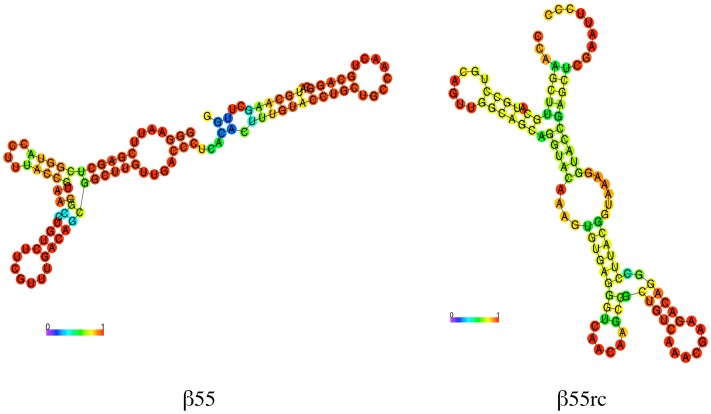
Predicted Secondary Structure of RNA Aptamers. Predicted secondary structure of the β55 (left) and β55rc (right) aptamer probes with base pair probability indicated by the color scale bar. Minimum free energy structures were determined using the RNAfold Webserver suite of programs.

The predicted secondary structures for the β55 aptamer and a RNA aptamer synthesized from the reverse complement of the β55 coding sequence (β55rc), shown in Figure 1, were determined using the RNAfold Webserver suite of programs (http://rna.tbi.univie.ac.at/cgi-bin/RNAfold.cgi) [40], [41]. The β55rc aptamer has a very different predicted secondary structure from that of β55 and was used as a control aptamer probe.

**Figure 2:**
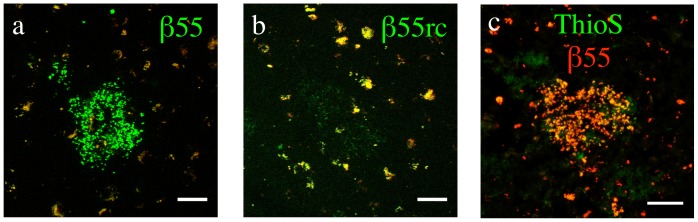
β55 Staining of Amyloid Plaques in Ex Vivo Human AD Brain Tissue. Merged red and green channel confocal images of frozen-section human AD brain tissue stained with biotinylated β55 (a) and β55rc (b). β55 positive plaques (green) were clearly visible, while only a few very faint β55rc positive plaques were observed. Background auto-fluorescence, observed in both red and green channels, is shown in yellow. (c) Fluorescence images of human AD brain tissue costained with biotinylated-β55 (red) and Thioflavin-S (green). β55 colocalized with Thioflavin-S positive plaques. (Scale bars: 50 µm).

The ability of the β55 aptamer to stain amyloid plaques was demonstrated by staining frozen-section brain tissue from AD subjects with biotinylated β55 and β55rc aptamers. While plaques were clearly visible with the β55 aptamer (Figure 2a), only a few very faint β55rc positive plaques were observed (Figure 2b). A tissue section co-stained with β55 and Thioflavin-S showed good colocalization of β55 and Thioflavin-S for amyloid plaques (Figure 2c).

**Figure 3:**
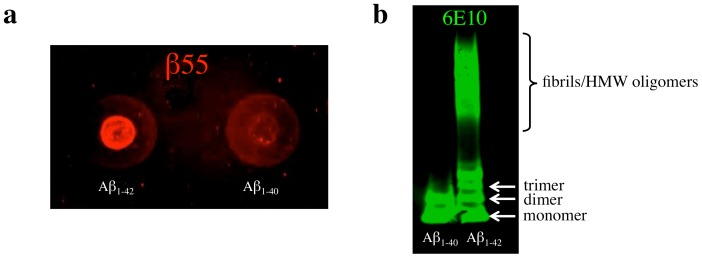
β55 Staining of Dot Blots of Synthetic Aβ Aggregates. (a) Dot blot of synthetic Aβ_1–42_ and Aβ_1–40_ aggregates probed with biotinylated-β55. (b) Western blot of the synthetic Aβ_1–42_ and Aβ_1–40_ aggregates probed with 6E10 antibody. The increased staining of Aβ_1–42_ aggregates in the dot blot relative to Aβ_1–40_ aggregates is consistent with the greater fibril and high molecular weight oligomer composition of Aβ_1–42_ aggregates observed in the western blot.

Dot blots of synthetic Aβ_1–40_ and Aβ_1–42_ that were incubated at 37°C for 3 days were performed to examine the binding of β55 to different Aβ species. β55 staining of both Aβ_1–40_ and Aβ_1–42_ was observed (Figure 3a). The increased staining observed for Aβ_1–42_ is consistent with the increased propensity of Aβ_1–42_ to form high molecular weight (HMW) species relative to Aβ_1–40_ as shown in the 6E10 western blot of the synthetic Aβ reactions (Figure 3b), where no HMW species were observed for Aβ_1–40_.

Sodium dodecyl sulfate (SDS) western blots of human AD brain homogenates were stained with both 6E10 monoclonal antibody, which is reactive to amino acid residues 3–8 of Aβ, and biotinylated β55 (Figure S1 and File S1). β55 and 6E10 bound many of the same bands, in particular higher molecular weight oligomer bands between 20 and 60 kDa. In contrast, no binding of monomeric Aβ or soluble APP (sAPP) by β55 was observed. While some caution must be taken in the interpretation of the western blot given the documented effects of SDS on Aβ oligomer structure and aggregation [42]–[46], the colocalization of 6E10 and β55 bands provides further support for the binding of β55 to Aβ oligomers.

**Figure 4:**
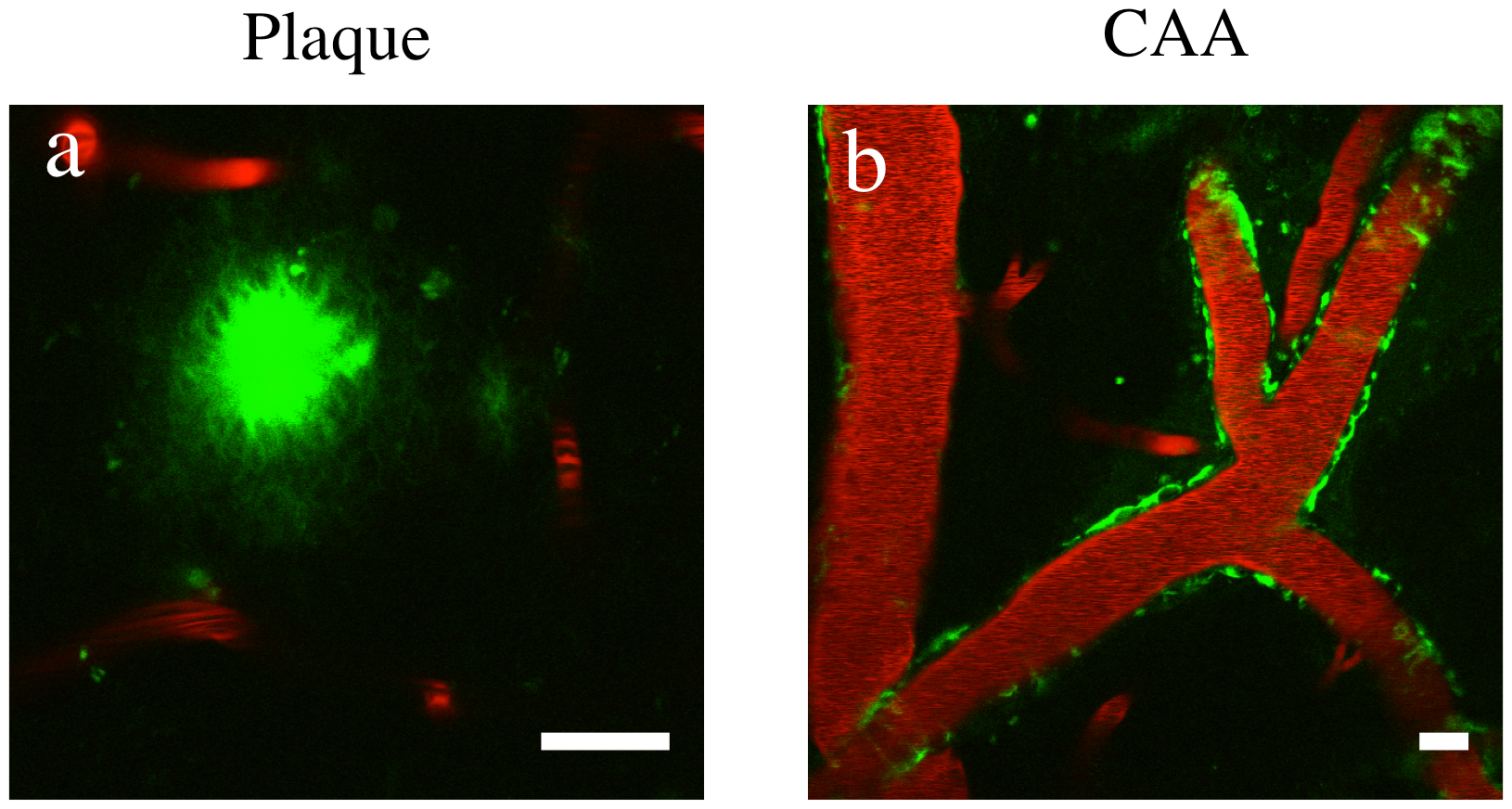
In Vivo Imaging of β55 Positive Amyloid Plaques. *In vivo* 2-photon microscopy images from an 18 month old APP/PS1 transgenic mouse obtained 1 hour after topical application of fluorescein-labeled β55 (a,b). Texas Red labeled dextran was intravenously injected for visualization of blood vessels. β55 positive plaques and cerebral amyloid angiopathy are clearly visible in the cortex (a) and vasculature (b), respectively. (Scale bars: 20 µm).

**Figure 5:**
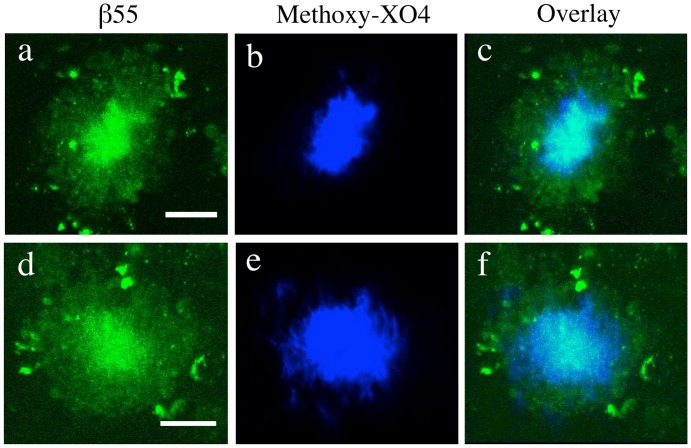
Colocalization of β55 and Methoxy-XO4 Positive Amyloid Plaques. *In vivo* 2-photon microscopy plaque images from a 7 month old APP/PS1 transgenic mouse acquired 1 hour after topical application of fluorescein-labeled β55 (a,d) and 1 day after intraperitoneal injection of methoxy-XO4 (b,e). While methoxy-XO4 stains only the dense core of the plaque, β55 stains both the plaque core and a diffuse halo surrounding the plaque (c,f). (Scale bars: 20 µm).

The ability of β55 to bind to plaques in post mortem tissue and fibrillar and non-fibrillar Aβ species on dot blots of synthetic Aβ raised the possibility that the aptamer may label plaques in an *in viv*o setting. *In vivo* 2-photon imaging studies performed in APP/PS1 transgenic mice, which overexpress mutant alleles of amyloid precursor protein (APPswe) and presenilin-1 (PS1dE9), showed that fluorescein-labeled β55 binds amyloid plaques in the cerebral cortex (Figure 4a) and vasculature (Figure 4b). Plaque staining by β55 was then compared to plaque staining with methoxy-XO4 (Figure 5). While methoxy-XO4 stained only the dense core of each plaque, β55 stained both the plaque core and a diffuse halo surrounding the plaque core (Figure 5c,f).

**Figure 6:**
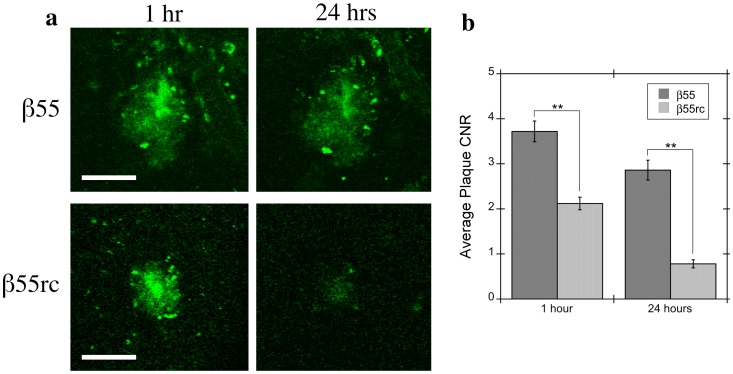
Contrast-to-Noise Ratio for β55 and β55rc Positive Amyloid Plaques. (a) Representative *in vivo* 2-photon microscopy images from 7.5 month old APP/PS1 transgenic mice acquired 1 hour (left column) and 24 hours (right column) after topical application of either fluorescein-labeled β55 (top row) or β55rc (bottom row). Most β55 plaques were still visible 24 hours after topical application. In contrast, only a small number of very faint β55rc plaques were still visible after 24 hours. (b) Average plaque contrast-to-noise ratio (CNR) observed 1 hour and 24 hours following topical application of fluorescein-labeled β55 (n = 2) or β55rc (n = 2). β55 positive plaques had a significantly greater CNR than β55rc plaques (p<0.01) at both time points. (Scale bars: 50 µm).

*In vivo* β55 plaque staining was compared to plaque staining with the β55rc control aptamer (Figure 6a). Images were acquired 1 and 24 hours after topical application of the aptamer probes in 2 mice for each aptamer. The β55 aptamer detected almost all of the methoxy-XO4 positive plaques at both 1-hour (96%) and 24-hour (83%) time points (see Table 2). In contrast, while the β55rc control aptamer detected 83% of plaques at the one-hour time point, only a few faint plaques (<30%) were detected after 24 hours. The contrast-to-noise-ratio (CNR) for β55 positive plaques was significantly greater (p<0.01) than that for β55rc plaques at both 1- and 24-hour time points (Table 2 and Figure 6b).

**Table 2:** Contrast-to-noise ratio (CNR) distribution of amyloid plaques observed in 2-photon *in vivo* images of 7.5 month-old APP/PS1 transgenic mice with β55 and β55rc.

	β55		β55rc	
	Time (hrs)		Time (hrs)	
CNR	1	24	1	24
0	4	22	20	84
1	20	17	52	19
2	17	18	24	12
3	16	9	13	4
4	16	16	6	0
5	6	8	0	0
6	8	4	1	0
7	4	3	1	0
8	0	1	0	0
9	2	1	2	0
10	1	0	0	0
11	1	0	0	0
Total plaques	99	99	119	119
Total detected	95	77	99	35
**% Detected**	**96.0**	**77.8**	**83.2**	**29.4**
**Average CNR**	**3.7±0.2**	**2.9±0.2**	**2.1±0.1**	**0.8±0.1**

To examine whether β55 induces plaque clearance or degradation, similar to antibody probes of amyloid plaques, longitudinal *in vivo* 2-photon imaging of a 10 month-old APP/PS1 mouse at 1 and 4 days following topical application of β55 was performed. However, no changes in plaque number or appearance were observed.

## Discussion

RNA aptamers have previously been shown to detect synthetic Aβ fibrils with very high sensitivity [35]–[37]. These RNA aptamers were selected in SELEX screens against either Aβ monomers or low molecular weight Aβ oligomers and may therefore prove to be excellent probes for detecting not only amyloid plaques, but also Aβ oligomers. Here we examined the ability of β55, an aptamer previously selected in a SELEX screen against synthetic Aβ_1–40_ [35], to bind oligomeric Aβ and amyloid plaques in *ex vivo* human AD brain tissue slices and in an *in vivo* transgenic mouse model of AD.

As demonstrated in Figure 2, robust staining of amyloid plaques by β55 was observed in *ex vivo* human AD brain tissue slices, while only a few very faint plaques were observed with the β55rc control aptamer. In addition, costaining with Thioflavin-S demonstrated that β55-positive plaques colocalize with Thioflavin-S stained plaques (Figure 2c). Similarly, β55 bound amyloid plaques in the cortex and vasculature of live APP/PS1 mice (Figure 4) and colocalized with methoxy-XO4 staining (Figure 5). Interestingly, β55 stained not only the plaque core, but also labeled a diffuse halo surrounding the core that was not observed with methoxy-XO4. These data suggest that β55 may bind smaller aggregates of Aβ surrounding the dense core, including oligomeric Aβ.

To gain more insight into whether β55 binds oligomeric Aβ, dot blots of synthetic Aβ aggregates were performed. Strong β55 staining of Aβ_1–42_ aggregates was observed with fainter staining of Aβ_1–40_ aggregates (Figure 3a). The difference in staining is attributed to the increased propensity of Aβ_1–42_ to form fibrils relative to Aβ_1–40_. This is demonstrated in the 6E10 western blot of the Aβ samples, where a large number of high molecular weight species were observed for Aβ_1–42_, but not Aβ_1–40_ (Figure 3b). The Aβ_1–40_ dot blot staining therefore indicates that β55 binds low molecular weight, non-fibrillar Aβ species. In accord with these findings, Tsukakoshi *et al* observed binding of a DNA aptamer to oligomeric Aβ_1–40_ [38].

The *in vivo* optical imaging data indicates that both β55 and β55rc control aptamer bind plaques. This is consistent with previous studies that demonstrated that RNA aptamers in general have a high affinity for amyloidigenic structures. In a SELEX screen for Aβ targeted RNA aptamers, Rahimi *et al* showed that both the naïve SELEX library and the final selected aptamers bound a large number of different amyloidogenic proteins, suggesting that RNA has an intrinsically high affinity for amyloidogenic, β-sheet structures [36]. Further support for the affinity of RNA for amyloidogenic structures is provided by a recent molecular modeling study that observed a very high homology between Aβ and the RNA binding protein AF-Sm1 [47] and by the fact that senile plaques have been found to contain high levels of RNA [48]–[50]. The affinity of RNA for amyloidogenic structures would explain the *in vivo* plaque binding observed for the β55rc aptamer that was used as a control in our study. However, while RNA may have some affinity for beta sheet structures, the fact that a significantly increased CNR was observed for β55 plaques compared to β55rc plaques in the *in vivo* multiphoton images (Figure 6 and Table 2), at both early and late imaging time points, suggests that there are differences in binding affinity with β55 having a significantly greater affinity than β55rc. This is consistent with the fact that only a few very faint, β55rc-positive plaques were visible in the *ex vivo* human AD brain tissue (Figure 2b). In addition, Ylera measured aptamer dissociation constants (K_d_) for binding to Aβ using affinity chromatography and while a K_d_ of 29 nM was determined for β55 no K_d_ was measureable for the RNA aptamer pool obtained from the first SELEX cycle [51], again indicating that RNA aptamers do display significant differences in binding affinity.

While the large molecular weight of the β55 aptamer (34 kDa) precludes its crossing the blood-brain barrier (BBB), a recent study by Cheng *et al*, in which an *in vivo* SELEX screen for a BBB penetrating aptamer was performed, did find an aptamer (A15) capable of crossing the BBB [10]. While the exact mechanism of the brain uptake of the BBB penetrating aptamer is unclear, given the large molecular weight of the aptamer (23 kDa) the most likely mechanism is by receptor-mediated transcytosis. Studies are currently underway to investigate the possibility of designing a fusion aptamer consisting of BBB penetrating and amyloid binding domains that would allow aptamers to be delivered non-invasively to the brain.

These studies demonstrate the great potential of RNA aptamers as *in vivo* and *ex vivo* imaging probes. One third of uracil nucleotides were fluorescein labeled, corresponding to a total of 10 fluoresceins per aptamer, providing high detection sensitivity. The incorporation of multiple fluorescein labels did not interfere with the amyloid binding. The fact that the fluorescein-labeled aptamer still bound plaques with high affinity is likely due to the fact that only a relatively small conserved region of the aptamer is required for binding. A significant fraction of the aptamer consists of sequences required for the selection/amplification process in the SELEX screen. In addition, topical application of β55 did not result in plaque clearance or degradation indicating that the aptamer is relatively inert biologically, in contrast to antibody probes that induce an inflammatory response and plaque clearance [32]. Finally, aptamers can be made with a variety of multimodal probes for optical, MR or PET imaging. Uracil labeled with a whole host of different optical tags is readily available and can easily be incorporated into the aptamer. In addition, cytosine and uracil nucleotides fluorinated at the 2′-pyramidine position, which are commercially available, can be used to make RNAse resistant aptamers that may also be detectable by ^19^F MRI. A previous study with a cold ^19^F PET probe specific for amyloid plaques was able to detect plaques using ^19^F MRI, however, with only a single ^19^F per probe molecule the sensitivity was very low and an extremely long MRI acquisition time of 90 minutes was required to obtain low resolution ^19^F images [52]. However, significantly greater ^19^F MRI sensitivity, and hence decreased signal acquisition times, would potentially be achievable with fluorinated β55, which contains 30 cytosine and 30 uracil nucleotides for a total of 60 ^19^F nuclei. Finally, a PET aptamer probe could be generated either by incorporating 5′-ethynyl-uridine, a commercially available uracil analog, into the RNA aptamer followed by click chemistry reaction with ^18^F-azide or by end labeling the aptamer with ^64^Cu.

## Conclusions

In summary, the β55 aptamer binds amyloid plaques in both *ex vivo* human AD brain tissue and *in vivo* APP/PS1 transgenic mice. Diffuse halos surrounding the methoxy-XO4 positive plaque cores were observed with β55 *in vivo*, which may represent oligomeric Aβ. The unprotected RNA aptamer appears to be quite stable and detectable for at least 24 hours under *in vivo* conditions when bound to amyloid plaques. In addition, no detectable tissue response to the aptamer application, such as plaque clearance, was detected. These data suggest that Aβ-targeted aptamers bind oligomeric Aβ and may be useful reagents for imaging both fibrillar and non-fibrillar Aβ. Moreover, these data illustrate the broader principle that RNA aptamers, which are nearly infinitely adaptable using SELEX technologies and that can be labeled with any number of off-the-shelf multimodal imaging agents (fluorophores, biotin, radioisotopes or epitope tags), can be developed with high enough detection sensitivity and selectivity to rival antibodies and small molecules in *in vivo* and *ex vivo* imaging studies.

## Supporting Information

Figure S1 **β55 Staining of Western Blot of Human AD Brain Tissue Extracts.** Western blot of human AD brain tissue extracts obtained after sequential treatment with TBS, 2% Triton X-100, 0.5% SDS, and 70% formic acid. The western blot was probed with both 6E10 antibody (green) and biotinylated-β55 (red). β55 binds many of the same bands as 6E10 (yellow asterisks). The green and red asterisks indicate bands unique to 6E10 or β55, respectively. Faint bands at ∼8 and 16 kDa were visible in the β55 image (arrows) of the SDS soluble fraction.
(TIF)

File S1 **Methods and Results for β55 Staining of Western Blot of Human AD Brain Tissue Extracts.**
(DOCX)
