# Supplementary figures and images for: RNA Aptamer Probes as Optical Imaging Agents for the Detection of Amyloid Plaques
Source: PLoS One. 2014 Feb 26;9(2):e89901. doi: 10.1371/journal.pone.0089901 (PMC3935954; doi:10.1371/journal.pone.0089901)

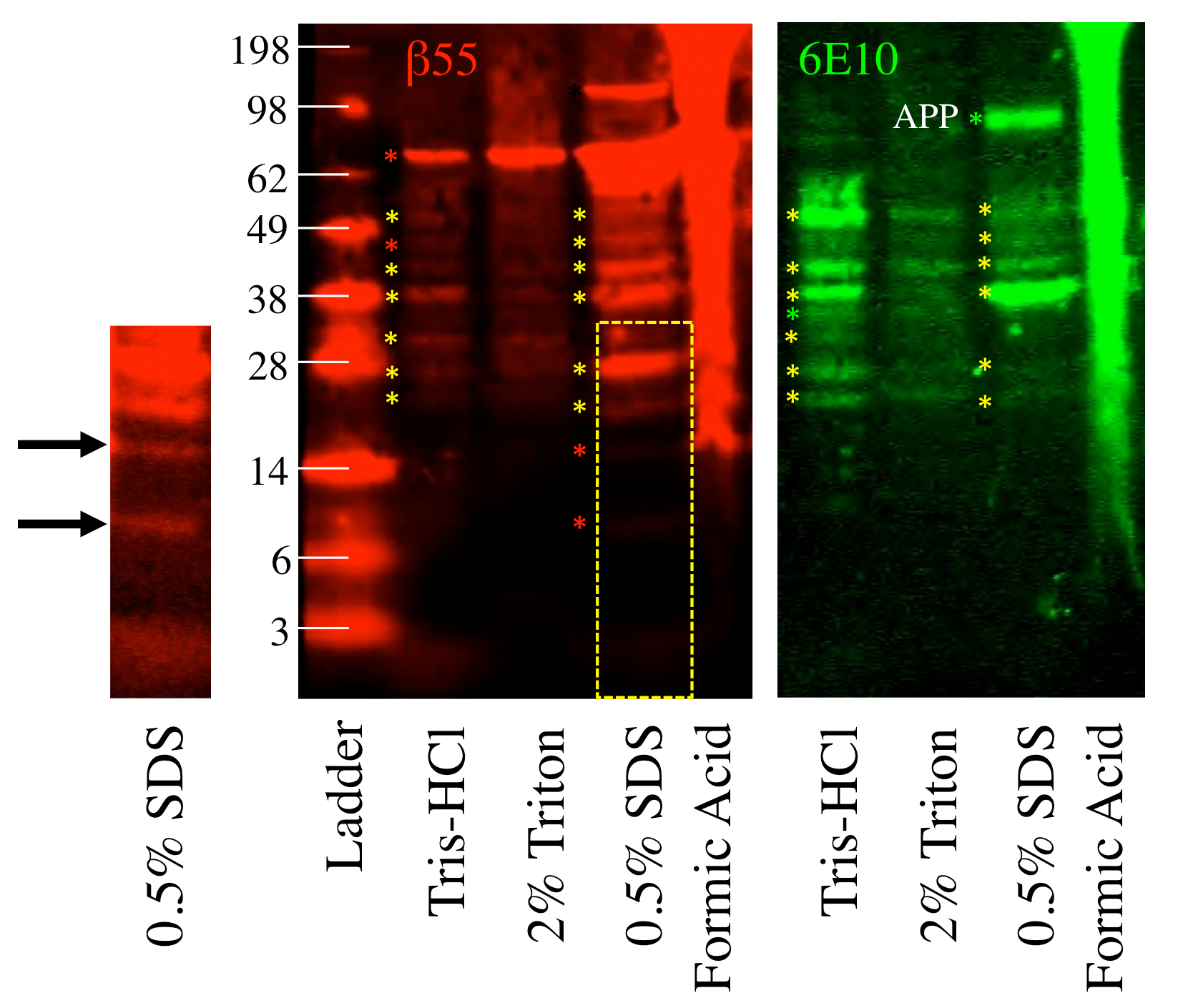

Supplement: Figure S1 — β55 Staining of Western Blot of Human AD Brain Tissue Extracts. Western blot of human AD brain tissue extracts obtained after sequential treatment with TBS, 2% Triton X-100, 0.5% SDS, and 70% formic acid. The western blot was probed with both 6E10 antibody (green) and biotinylated-β55 (red). β55 binds many of the same bands as 6E10 (yellow asterisks). The green and red asterisks indicate bands unique to 6E10 or β55, respectively. Faint bands at ∼8 and 16 kDa were visible in the β55 image (arrows) of the SDS soluble fraction. (TIF) [file pone.0089901.s001.tif]
