# Supplementary material for: RNA Aptamer Probes as Optical Imaging Agents for the Detection of Amyloid Plaques
Source: PLoS One. 2014 Feb 26;9(2):e89901. doi: 10.1371/journal.pone.0089901 (PMC3935954; doi:10.1371/journal.pone.0089901)
Supplement: File S1 — Methods and Results for β55 Staining of Western Blot of Human AD Brain Tissue Extracts. (DOCX) [file pone.0089901.s002.docx]

**Supporting Information**

**Western blot of human AD brain tissue homogenates**

A western blot of human Alzheimer’s disease (AD) brain homogenates was performed to gain insight into which plaque components are bound by the β55 aptamer (Figure S1). Protein fractions were extracted from frozen-section AD brain tissue following serial homogenization with Tris-buffered saline (TBS), 2% Triton X-100 in TBS, 0.5% sodium dodecyl sulfate (SDS) in TBS, and 70% formic acid. A western blot of the different protein fractions was stained with both 6E10 antibody (1:1000), which is reactive to amino acid residues 3-8 of Aβ, and biotinylated β55. Probes were visualized by secondary staining with streptavidin IRDye 700DX (Rockland Immunochemicals, Gilbertsville, PA) and anti-mouse IgG antibody conjugated to IRDye800 (Rockland Immunochemicals, Gilbertsville, PA) and imaged on an Odyssey infrared imaging system (LI-COR Biosciences, Lincoln, NB).

β55 and 6E10 bound many of the same bands, in particular higher molecular weight bands between 20 and 60 kDa (Figure S1). Two β55 positive bands observed at approximately 8 and 16 kDa in the SDS soluble fraction might be attributable to low molecular weight Aβ oligomers (dimers, trimers and/or tetramers), however, no 6E10 staining of these bands was observed. The high molecular weight smear in the formic acid soluble fraction was observed with both 6E10 and β55. While the 6E10 smear band extended from Aβ monomer or dimer to high molecular weight (HMW) fibrils, strikingly, the β55 aptamer smear band extended only from the 14 kDa band (Aβ trimer or tetramer) to HMW fibrils. This suggests that the β55 aptamer did not bind to monomeric Aβ.  Finally, no evidence of β55 binding soluble amyloid precursor protein (sAPP) was observed.
